# Scientometric analysis of research on trachoma in Brazil, 2000–2020

**DOI:** 10.11606/s1518-8787.2022056004144

**Published:** 2022-11-18

**Authors:** Adjoane Maurício Silva Maciel, Alberto Novaes Ramos, Anderson Fuentes Ferreira, Nádia Maria Girão Saraiva de Almeida, Vivian da Silva Gomes, Daniela Vaz Ferreira Gómez, Roberto da Justa Pires

**Affiliations:** I Universidade Federal do Ceará Faculdade de Medicina Fortaleza CE Brasil Universidade Federal do Ceará. Faculdade de Medicina. Programa de Pós-Graduação em Saúde Pública. Fortaleza, CE, Brasil; II Secretaria Municipal de Saúde Russas CE Brasil Secretaria Municipal de Saúde. Russas, CE, Brasil; III Universidade Federal do Ceará Faculdade de Medicina Departamento de Saúde Comunitária Fortaleza CE Brasil Universidade Federal do Ceará. Faculdade de Medicina. Departamento de Saúde Comunitária. Fortaleza, CE, Brasil; IV Universidade Estadual do Ceará Mestrado Profissional em Saúde da Criança e do Adolescente Fortaleza CE Brasil Universidade Estadual do Ceará. Mestrado Profissional em Saúde da Criança e do Adolescente. Fortaleza, CE, Brasil; V Saúde do Estado do Ceará Fortaleza CE Brasil Secretaria de Saúde do Estado do Ceará. Fortaleza, CE, Brasil; VI Ministério da Saúde Secretaria de Vigilância em Saúde Brasília DF Brasil Ministério da Saúde. Secretaria de Vigilância em Saúde. Brasília, DF, Brasil

**Keywords:** Trachoma, Scientific and Technical Publications, Citation Databases, Bibliometrics, Bibliometric Indicators

## Abstract

**OBJECTIVE:**

To analyze the scientometric profile of research on trachoma in Brazil.

**METHODS:**

Bibliographic research of publications on trachoma in Brazil indexed by the Scopus database from 2000 to 2020, based on specific criteria. Data on authorship, country of origin, institutions, and keywords were collected and analyzed with analysis of time trends. Bibliographic networks were constructed via a scientometric visualization software—VOSviewer^®^ 1.6.16.

**RESULTS:**

We analyzed 42 publications on trachoma in Brazil. The annual average was two articles, with an increase of about 50% during the period. The average number of authors was three per document and school surveys were the most common subject category. Most published articles came from Brazilian institutions (95.2%), mainly those based in Southeast and North Brazil. Of the most productive authors, 10 were mentioned as first author in 26.2% of publications (11/42) and the predominant institutions are based in the state of São Paulo. The term “trachoma” (n = 18) was the most recurrent keyword.

**CONCLUSION:**

This first scientometric analysis of research on trachoma in Brazil showed a limited number of studies on this disease. The scientific production slightly increased, although the origin of many studies is geographical areas with lower endemicity of this disease. Greater investments are needed for a better understanding and control of this neglected tropical disease. The analysis of bibliographic production on this topic is important to strengthen the development of research and strategic planning of programs for the control of trachoma and neglected tropical diseases in general.

## INTRODUCTION

Trachoma is a neglected tropical disease (NTD)^1^ caused by the bacterium *Chlamydia trachomatis* and critically affects people's health by chronic and associated recurrent keratoconjunctivitis^2,3^. In the world, it is the main cause of blindness of infectious origin^1^.

Its high morbidity is associated with poverty in different contexts of vulnerability, such as low schooling level, lack of basic sanitation and hygiene, and restriction on access to health services^1^. Its occurrence, therefore, prevents carriers of the disease from having a good eye health and affects their overall well-being, functional capacity, social inclusion, and quality of life^4^.

In 2019, 142.2 million people lived in endemic areas for trachoma worldwide^3^ and 1.9 million of them at risk of evolution to visual impairment or irreversible blindness^5^.

Until 2020, nine countries in the world had the elimination of trachoma as a public health problem^6^. In at least 44 countries, this disease is recognized as a public health problem. In Latin America, it persists in different areas of Brazil, Colombia, Guatemala, and Peru, and Mexico validates its elimination^1^.

The first stage of the *Inquérito Nacional para Validação da Eliminação do Tracoma como Problema de Saúde Pública* (Survey for Validation of Elimination of Trachoma as a Public Health Problem), performed in Brazil in from 2018 to 2019, showed that the prevalence of the active form of the disease (follicular trachoma) was below 5% among children from one to nine years of age in non-indigenous areas. The sequelar form of the disease (trachomatous trichiasis), which is unknown by the health system, was lower than the critical value of 0.2%, except for the Northeast Ceará evaluation unit, which presented a prevalence of 0.22%^7^; however, this value was included in the confidence interval.

In line with the agenda to achieve the Sustainable Development Goals (SDG), the World Health Organization proposes the elimination of trachoma as a public health problem in the world as one of the specific targets for NTD from 2021 to 2030^8^. Eye health is considered essential to achieve many of the SDG^4^.

Although Brazil continues to be a priority area for trachoma control^1^, the number of scientific publications considering the epidemiological context and different patterns of endemicity in states and municipalities is still limited^1^. Identifying patterns of production of studies on trachoma in Brazil is a strategy to support researchers, governments, and funding agencies to make more efficient decisions.

Scientometric analyses show patterns of scientific production and support researchers, governments, and funding agencies to identify areas and topics with little investment (such as trachoma) and make more efficient decisions. The trend of use of this research method is increasing, including in Brazil, which enables the recognition of research efforts from the quantitative description of documents^9^, scientific collaboration, and the characterization of scientific social networks related to the topic^10^.

Thus, this study aims to develop the first scientometric analysis on trachoma in Brazil, analyzing the profile of research on the topic from 2000 to 2020 in order to generate subsidies for the proposal of actions and policies on the research, surveillance, and control of trachoma in the country.

## METHODS

This is a scientometric research on publications related to trachoma in Brazil, which used scientific productions from this country or epidemiological data related to the disease.

Data were collected in July 2021 from all publications from January 2000 to December 2020, totaling a 21-year analysis series.

The scientific productions analyzed were indexed in the Scopus (https://www.scopus.com/home.uri), PubMed (https://pubmed.ncbi.nlm.nih.gov/), Web of Science (https://mjl.clarivate.com/search-results), and Dimensions (https://app.dimensions.ai/discover/publication) databases and were accessed by the C*omunidade Acadêmica Federada da Coordenação de Aperfeiçoamento de Pessoal de Nível Superior* (CAFe-CAPES – Federated Academic Community of the Coordination of Superior Level Staff Improvement). All data presented analytical compatibility in the scientometric visualization software—VOSviewer^®^ 1.6.16 (https://www.vosviewer.com/). This software can be used to build a scientific knowledge network and shows research structure, evolution, and cooperation^11^.

Based on specific criteria, an advanced search was performed with keywords. In the Scopus database, the following terms were searched: *((AUTHKEY (“Trachoma”) OR TITLE (“Trachoma”) OR ABS (“Trachoma”))* and *(AUTHKEY (“Brazil”) OR TITLE (“Brazil”) OR ABS (“Brazil”)) OR (AUTHKEY (“Tracoma”) OR TITLE (“Tracoma”) OR ABS (“Tracoma”)) OR (AUTHKEY (“Brasil”) OR TITLE (“Brasil”) OR ABS (“Brasil”))).* In PubMed: *(((tracoma[MeSH Terms]) OR (Tracoma[Title])) OR (Tracoma[Title/Abstract]))* and *(((Brasil[MeSH Terms]) OR (Brasil[Title])) OR (Brasil[Title/Abstract]))) OR (((trachoma[MeSH Terms]) OR (Trachoma[Title])) OR (Trachoma[Title/Abstract]))* and *(((Brazil[MeSH Terms]) OR (Brazil[Title])) OR (Brazil[Title/Abstract])))*. In Web of Science: *((TI=(trachoma) OR AB=(trachoma) OR AK=(trachoma)) AND (TI=(Brazil) OR AB=(Brazil) OR AK=(Brazil))) OR ((TI=(tracoma) OR AB=(tracoma) OR AK=(tracoma))* and *(TI=(Brasil) OR AB=(Brasil) OR AK=(Brasil)))]*. And, finally, in Dimensions: *(trachoma and Brazil) OR (tracoma and Brasil)]*.

The Scopus database was selected for the analysis of indicators and scientometric relationships due to its greater number of documents used, the availability of variables, and VOSviewer^®^ specificities. However, certain periods of time presented limitations due to issues related to the indexation of some important national and international journals, as well as expert opinions.

Scientometric analysis indicators related to co-authorship were used for the analysis units “author” (co-authorship *versus* author), “institution” (co-authorship *versus* organizations), and “country” (co-authorship *versus* countries), and for the co-occurrence for the unit “author keywords” (co-occurrence *versus* author keywords), following parameters with maximum limit of ‘25’ and minimum of ‘1’ for scientometric visualization items.

In the unit “author”, the authors of the selected studies were analyzed within the period. The unit “institution” is related to the affiliation of each author and “country” refers to the nationality of the institutions of the authors. For the presentation of the results, the 10 highest occurrences of each analysis unit were highlighted and presented.

For structuring bibliographic networks, the specificities of references associated with each record were considered along with author keywords data. The most frequent terms in publications and cluster analysis were provided by VOSviewer^11^.

Then, images representing the relationships (maps) between authors, countries, institutions, and keywords (nodes), the strength between these relationships (arc thickness), and the number of their total contributions (node size) were observed. A thesaurus was also applied to consolidate the analyzed terms.

This study was approved by the Research Ethics Committee of the *Hospital São José de Doenças Infecciosas* (HSJ)/Ceará Health Department, under Opinion No. 3,634,979 (CAAE 18663119.7.0000.5044).

## RESULTS

### Scientific Literature, Spatial Distribution, and Time Trends

Initially, after excluding duplicates, we selected scientific publications in the Scopus (identified: 59; selected: 42), Dimensions (identified: 52; selected: 44), PubMed (identified: 34; selected: 29), and Web of Science (identified: 31; selected: 26) databases during the period studied. We identified 52 publications in common to all databases (Table 1).

**Table 1:** Characterization of studies on trachoma according to title, author, year of publication, study location, and database. Brazil, 2000–2020 (n = 52).

n	Title	Author	Year of publication	Study location	Database			
					Scopus^a^	Dimensions	PubMed	Web of Science
1	Chlamydia trachomatis serotype A infections in the Amazon region of Brazil: prevalence, entry and dissemination	Ishaket al.^23^	2015	Maranhão, Pará e Amapá (states)	X	X	X	X
2	Community-based transconjunctival marginal rotation for cicatricial trachoma in Indians from the Upper Rio Negro basin	Soares et al.^17^	2004	Região Alto Rio Negro - Amazonas	X	X	X	X
3	Corneal findings not related to entropion or trichiasis after trachoma	Chaves et al.	2001	São Paulo (state)	X			
4	Diseases Neglected by the Media in Espírito Santo, Brazil in 2011–2012	Cavaca et al.^22^	2016	Espírito Santo (state)	X	X	X	X
5	Elaboration and validity and reliability analysis of a questionnaire to assess the knowledge of primary care physicians and nurses about trachoma	Silva et al.	2020	-	X			
6	Epidemiologic study of trachoma in a community of “Chapada do Araripe”, Pernambuco State - Brazil	Lucena et al.	2004	Ipubi - Pernambuco	X			
7	Epidemiological study of trachoma (letter)	Mörschbächer et al.	2011	-	X			
8	Epidemiology of trachoma in the village of Araripe plateau - Ceará State	Lucena et al.^24^	2010	Porteiras - Ceará	X	X	X	X
9	Factors associated with trachoma in a low-endemic area in southeast Brazil	D’Amaral et al.	2005	São Paulo - São Paulo	X	X	X	
10	Factors associated with trachoma treatment and control treatment in schools of municipality of the Northeast Region, Brazil	Maciel et al.^27^	2020	Russas - Ceará	X	X	X	
11	Household Survey of Trachoma among Children Living in Pernambuco, Brazil	Brito et al.^14^	2019	Pernambuco (state)	X	X	X	X
12	It was urgent and indispensable to act: The trachoma in São Paulo in the early twentieth century	Lódola et al.	2019	-	X			
13	Laboratory diagnosis of trachoma in Serrolândia village of Ipubi Town, Pernambuco – Brazil	Lucena et al.	2005	Ipubi - Pernambuco	X			
14	Preliminary evidence that synanthropic flies contribute to the transmission of trachoma causing Chlamydia trachomatis in Latin America	Reilly et al.	2007	Ilha de Marajó - Pará	X	X	X	X
15	Prevalence and spatial distribution of trachoma among schoolchildren in Botucatu, São Paulo – Brazil	Schellini et al.	2010	Botucatu - São Paulo	X	X	X	X
16	Prevalence of infection by Chlamydia trachomatis in ocular samples of patients with conjunctivitis in genetic and molecular biology laboratory from metropolitan area of Florianópolis, Brazil	Machado et al.	2009	Florianópolis - Santa Catarina	X			X
17	Prevalence of trachoma and associated factors in students from the Jequitinhonha Valley, Minas Gerais, Brazil	Silva et al.	2020	Vale do Jequitinhonha - Minas Gerais	X	X	X	X
18	Prevalence of trachoma among schoolchildren in Bauru - São Paulo State, Brazil	Ferraz et al.	2010	Bauru - São Paulo	X	X	X	X
19	Prevalence of trachoma in a population of the upper Rio Negro basin and risk factors for active disease	Cruz et al.	2008	São Gabriel da Cachoeira - Amazonas	X	X	X	X
20	Prevalence of trachoma in Botucatu city - Sao Paulo state [Prevalência de tracoma cicatricial em Botucatu - Estado de São Paulo]	Schellini et al.	2006	Botucatu - São Paulo	X			
21	Prevalence of trachoma in Brazilian schoolchildren	Lopes et al.	2013	Brasil (municipalities)	X	X	X	X
22	Prevalence of trachoma in preschool and schoolchildren in the city of São Paulo	Koizumi et al.	2005	São Paulo - São Paulo	X	X	X	
23	Prevalence of trachoma in schoolchildren in Brazil	Luna et al.	2016	Brasil (municipalities)	X	X	X	X
24	Prevalence of trachoma in schoolchildren in the Marajo Archipelago, Brazilian Amazon, and the impact of the introduction of educational and preventive measures on the disease over eight years	Favacho et al.	2018	Ilha de Marajó - Pará	X	X	X	X
25	Prophylaxis and treatment of diseases in western São Paulo state: the Sanitation Service and trachoma in the early twentieth century	Lodola et al.	2020	-	X	X	X	
26	Sequelae from Epidemic Viral Conjunctivitis Can Be Associated with Inflammatory Trachoma in Schoolchildren?	Meneghim et al.	2016	Botucatu - São Paulo	X	X	X	X
27	Spatial distribution of trachoma cases in the City of Bauru, State of São Paulo, Brazil, detected in 2006: defining key areas for improvement of health resources	Macharelli et al.^29^	2013	Bauru - São Paulo	X	X	X	X
28	Survey of trachoma within school students in the state of Roraima, Brazil	Medina et al.	2011	Roraima (state)	X	X	X	X
29	Trachoma among the Yanomami Indians	Paula et al.	2002	Região Alto Rio Negro - Amazonas	X	X	X	X
30	Trachoma and corneal diseases among Indians of the Alto Rio Negro, Amazonas, Brazil	Reis et al.	2002	Região Alto Rio Negro - Amazonas	X	X		
31	Trachoma and ethnic diversity in the Upper Rio Negro Basin of Amazonas State, Brazil	Alves et al.	2002	Região Alto Rio Negro - Amazonas	X	X	X	X
32	Tracoma: de lo básico a lo clínico	Carvajal-Fernández et al.	2017	-	X			X
33	Trachoma: Epidemiologic study of scholars from Alagoas state-Brazil	Damasceno et al.	2009	Alagoas (state)	X	X	X	X
34	Trachoma epidemiological school survey in the city of Embu das Artes – SP	Caninéo et al.	2012	Embu das Artes - São Paulo	X	X	X	
35	Trachoma elimination in Latin America: prioritization of municipalities for surveillance activities	Saboyá-Díaz et al.^1^	2019	América Latina	X	X	X	X
36	Trachoma in Indigenous Settlements in Brazil, 2000–2008	Freitas et al.	2016	Brasil	X	X	X	X
37	Trachoma in patients with allergic conjunctivitis	Bezerra et al.	2010	João Pessoa - Paraíba	X			
38	Trachoma in schoolchildren of the city of Botucatu, Sao Paulo, Brazil: detection and health promotion of a neglected disease	Meneghim et al.^25^	2016	Botucatu - São Paulo	X			
39	Trachoma prevalence among schoolchildren in the municipality of Turmalina, Minas Gerais state	Silva et al.	2016	Turmalina - Minas Gerais	X			
40	Trachoma prevalence and risk factors among preschool children in a central area of the city of São Paulo, Brazil	Caligaris et al.	2006	São Paulo - São Paulo	X	X	X	X
41	Trachoma prevalence in preschoolers and schoolchildren in Botucatu, São Paulo State, Brazil,1992	Medina et al.^21^	2002	Botucatu - São Paulo	X	X	X	
42	Trachoma: Still being an important blinding disease (review)	Schellini et al.	2012	-	X			X
43	Analysis of interventions and socio environmental factors associated with the occurrence of trachoma in Pernambuco in two surveys on schoolchildren conducted in 2006 and 2012	Alves et al.	2016	Pernambuco (municipalities)		X		
44	Blinding trachoma among Maku Indians of the upper Rio Negro: a neglected public health problem	Cruz et al.	2017	Região Alto Rio Negro - Amazonas			X	
45	Elimination of Trachoma as cause of blindness in Itapevi, State of Sao Paulo, Brazil	Joseph et al.	2015	Itapevi - São Paulo				X
46	Epidemiological aspects and prospects of the elimination of Trachoma (2018-2020) as a public health problem in Brazil	Gómez et al.	2018	Brasil (states)		X		
47	Epidemiology and operational aspects of trachoma surveillance and control in a school in the Municipality of São Paulo, Brazil	Chinen et al.	2006	São Paulo - São Paulo		X		
48	Estratégia eficaz para o enfrentamento do tracoma no Estado do Ceará	Gomes et al.	2019	Fortaleza - Ceará		X		
49	Epidemiological survey about of trachoma in students schools belonging to cities localized in the IV Gerencia Regional de Saude located in the state of Pernambuco, Caruaru –PE.	Germinio et al.	2016	Caruaru - Pernambuco		X		
50	Survey of prevalence trachoma of children in Distrito Federal, Brazil, july/2010	Jesus et al.	2013	Distrito Federal- Brasília		X		
51	Trachoma as cause of blindness: literature review	Silva et al.	2017	-		X		
52	Tracoma: uma antiga patologia ainda negligenciada na atualidade	Silva et al.	2015	-		X		

Source: Scopus, PubMed, Web of Science, Dimensions.

a Scopus database with analyzed publications from n = 1 to n = 42.

Review article, editorial (gray literature).

For analysis, we considered 42 scientific publications from the Scopus database and 138 authors. The scientometric analysis software used (VOSviewer^®^) processes one database in its routine (Table 1). The typology “original article” was the main type of publication, as it represented 95.2% (40/42) of all studies analyzed (Table 1).

Surveys among schoolchildren (n = 14), analysis in indigenous populations (n = 4), sequelae of the disease (n = 4), laboratory diagnoses (n = 4), trachoma risk factors (n = 3), “gray literature” (n = 6), population-based surveys (n = 2), spatial distribution (n = 1), epidemiological categorization of municipalities for surveillance purposes (n = 1), and other subjects (n = 3) are among the most common study topics.

**Figure 1:**
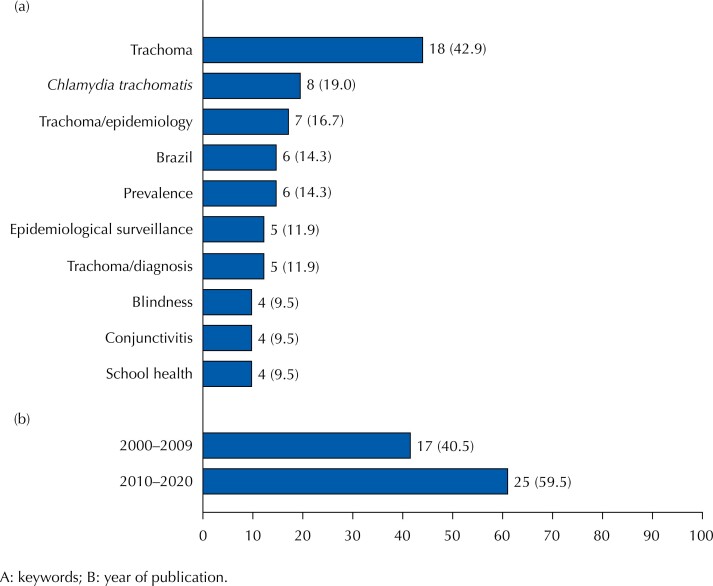
Bibliographic production on trachoma. Brazil, 2000–2020 (n = 42).

We observed bibliographic production in all regions of Brazil and a general increase of 50% in it during the second decade of this study (2010–2020) (Figure 1).

We found studies on trachoma in 13 Brazilian states: São Paulo (n = 12), Amazonas (n = 5), Pernambuco (n = 3), Ceará (n = 2), Minas Gerais (n = 2), Alagoas (n = 1), Espírito Santo (n = 1), Maranhão, Amapá (n = 1), Pará (n = 3), Paraíba (n = 1), Roraima (n = 1), and Santa Catarina (n = 1). We also found a representation of general data of Brazil (n = 3) and Latin America (n = 1). We considered other studies “gray literature” (n = 6) (Table 1).

### Scientometric Analysis

The scientometric analysis on trachoma showed an annual average of two articles during the last two decades in Brazil. The average number of authors were three per document and ranged from two to 10 authors for each publication (Table 1).

**Figure 2:**
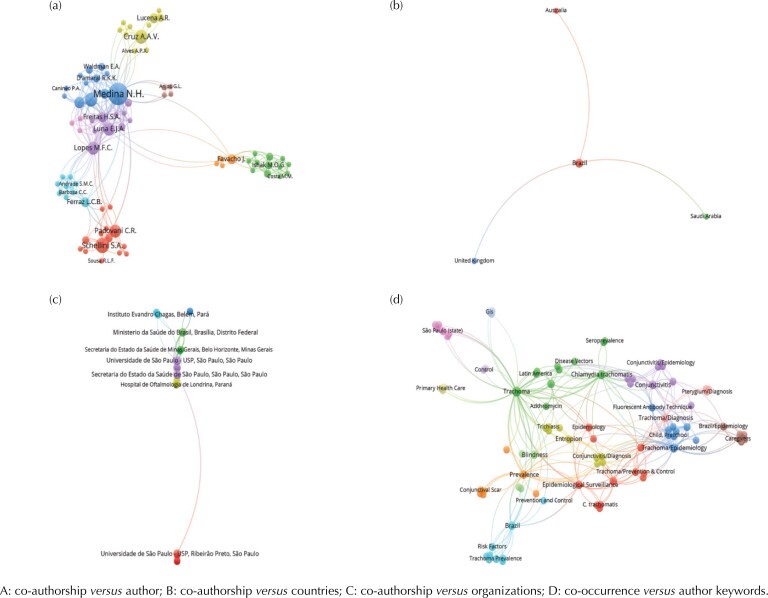
Relationships in the bibliographic production on trachoma. Brazil, 2000–2020 (n = 42).

The 10 most productive researchers were the first author in 26.2% (11/42) of publications. Medina N.H. presented greater contributions, as she participated in 35.7% (15/42) of scientific publications. Cruz A.A.V. is proportionally the most cited author (Table 2). In the relationship *“*co-authorship *versus* author,” the productivity of Medina N.H. (blue cluster) and the connection with other clusters stand out, followed by the scientific production network of the authors Schellini S.A. (pink cluster), Cruz A.A.V. (green cluster), and Cardoso M.R.A. (blue cluster) (Figure 2A).

**Table 2:** Bibliographic production on trachoma according to author and country. Brazil, 2000–2020 (n = 42).

Bibliographic production	Number of publications	Betweenness centrality		Productivity (%)
		Citations	Bond strengh	
Author (Open Researcher and Contributor ID [ORCiD])				
Medina, N.H. (0000-0002-6544-6674)	15	142	70	35.7
Schellini, S.A. (0000-0002-6938-1230)	7	20	23	16.7
Cruz, A.A.V. (0000-0002-8972-5571)	6	77	14	14.2
Cardoso, M.R.A. (0000-0001-6092-9215)	6	47	34	14.2
Lopes, M.F.C. (0000-0002-5392-1001)	5	28	37	11.9
Luna, E.J.A. (0000-0002-1145-9672)	5	27	30	11.9
Padovani, C.R. (0000-0002-7719-9682)	5	12	19	11.9
Koizumi, I.K. (0000-0001-5602-4878)	4	30	26	9.5
Caligaris, L.S.A. (não encontrado)	3	29	19	7.1
Favacho, J. (0000-0001-7926-7952)	3	23	14	7.1
Country (Institution)				
Brazil	40	211	3	95.2
United Kingdom	1	12	1	2.4
Australia	1	4	1	2.4
Mexico	1	1	1	2.4
United States	1	1	1	2.4
Saudi Arabia	1	0	1	2.4
Colombia	1	0	0	2.4

Source: VOSviewer®.

Studies on trachoma in Brazil were from institutions from seven countries. We identified publications by authors from institutions in Brazil (n = 40) and simultaneously in the United Kingdom (n = 1), Australia (n = 1), and Saudi Arabia (n = 1). Publications of authors exclusively outside the country came from institutions in the United States and Mexico (n = 1) and Colombia (n = 1) (Table 2). The analysis of “co-authorship *versus* countries” showed the relationship between publications and institutions from the Americas (n = 4), Europe (n = 1), Asia (n = 1), and Oceania (n = 1) (Figure 2B).

In total, scientific publications included 55 institutions, with a predominance of the 10 most productive from the state of São Paulo (6/10; 60%) and Southeast Brazil and more expressive participation of the São Paulo Health Department (33.3%) and the Universidade de São Paulo (USP) (26.2%) (Table 3). The relationship “co-authorship *versus* organizations” stands out the relationship between institutions from the Southeast (São Paulo) and North (Amazonas) Brazil and the Ministry of Health, which acts as a connection point with other states (Figure 2C).

**Table 3:** Bibliographic production on trachoma according to institution. Brazil, 2000–2020, (n = 42).

Institution	Number of publications	Publications (%)	Citations
Secretaria do Estado da Saúde de São Paulo, São Paulo, São Paulo	14	33.3	112
Universidade de São Paulo (USP), São Paulo, São Paulo	11	26.2	80
Universidade Estadual Paulista (UNESP), Botucatu, São Paulo	7	16.7	20
Universidade de São Paulo (USP), Ribeirão Preto, São Paulo	5	11.9	51
Ministério da Saúde do Brasil, Brasília, Distrito Federal	5	11.9	28
Instituto Evandro Chagas, Belém, Pará	3	7.1	23
Universidade Federal de São Paulo (UNIFESP), São Paulo, São Paulo	3	7.1	16
Universidade Estadual de Montes Claros, Montes Claros, Minas Gerais	3	7.1	1
Universidade Federal dos Vales do Jequitinhonha e Mucuri, Diamantina, Minas Gerais	3	7.1	1
Secretaria Municipal de Saúde de São Paulo, São Paulo	2	4.8	23

Source: VOSviewer®.

Note: selection/classification: 1. Number of publications; 2. Citations.

We identified the 10 keywords most frequently mentioned in the scientific publications analyzed. The term “trachoma” (n = 18) was the most frequent keyword—twice as frequent as “*Chlamydia trachomatis*” (n = 8) and “trachoma/epidemiology” (n = 7) (Figure 1). The analysis of “co-occurrence *versus* author keywords” showed a greater relationship between the keywords aforementioned (Figure 2D).

## DISCUSSION

This unprecedented study proves the limited number of studies on trachoma in Brazil and shows that most of them were performed by researchers outside the main endemic areas of Brazil. Despite the increasing trend observed during the last decade, critical gaps still persist, restating the character of neglected disease^9^ not only because of failures of science, but failures of public policies^12^.

Although the Global Trachoma Mapping Project encourages research worldwide to define the baseline trachoma map in endemic countries^13^, the insufficient number of research on trachoma in Brazil reduces the recognition of the disease, as well as the planning and implementation of control actions^14^. This context indirectly points to low prioritization of investments in research on the topic to overcome this chronic condition in endemic countries^15^, as the limitations highlighted by this study showed.

Brazil represents a recognized scientific leadership in topics of tropical medicine, especially in Latin America, with a remarkable contribution to several NTD^1^, however, the number of publications on topics related to trachoma is limited.

Eye health and vision have important general implications for various dimensions of life, health, sustainable development, and economics^4^. These aspects significantly hinder the achievement of the SDG, especially the goal of eliminating the disease by 2030. They restate the Brazil's responsibility as an endemic country to ensure additional investments to intensify surveillance and control actions^15^ and to establish strategies based on consistent partnerships for technological and scientific development^16^.

Thus, eye health is essential to achieve many of the SDG by 2030. Since the 1990s, the estimated prevalence of onchocerciasis and trachoma, the major infectious causes of blindness, decreased significantly. By 2030, the onchocerciasis transmission is expected to finally stop and trachoma to be eliminated as a public health problem in all countries worldwide^4^.

Scientific production on trachoma is significantly concentrated in North and Southeast Brazil, so that São Paulo and Amazonas are the most prominent federative units. This distribution, in regard to North Brazil, may be linked to the production of research on trachoma with indigenous populations in endemic contexts^17^.

However, research in this region was more frequent in the past decade, which shows the need to sustain the capacity to develop research focused on more vulnerable populations in endemic areas^15^.

The most participatory institutions in bibliographic production are from Southeast Brazil, especially São Paulo, which is probably associated with the greater funding, infrastructure, and research development capacity of these institutions^18^. We can also consider the capacity of promoting technical and scientific cooperation between groups of researchers from Brazil and abroad^19^.

The origin of the publications shows a critical contrast in the production on trachoma in institutions from geographical areas with lower endemicity of this disease^16^. The existence of research is directly related to human and social development. Moreover, access to specific funding for research on NTD, especially those more neglected, such as trachoma^15^, is limited.

The mobilizing power of authors for a topic and the collaboration between them, which are indispensable elements for scientific production capacity^20^, are other important aspects. However, almost in the two decades of this analysis, the average number of authors of research on trachoma slightly increased and it reinforces the lack of prioritization. Greater investments in scientific cooperation can expand integration with institutions in areas that present less research development^20^. However, this process can cause dependence on the definition of topics, without considering the needs of the places where the disease affects the most^16^.

Researchers with first authorship are important to promote collaboration between other authors. Besides having a considerable scientific production, those researchers establish important links and cooperate in conducting studies in institutions with possible access to research funding^16^. Thus, they act as an important link to construct the bibliographic networks analyzed in this study^10^. Despite the increase, the cooperation between researchers on trachoma in Brazil still shows a relative distance between authors of studies not linked to institutions with more recurrent affiliation in publications and co-authorship. It also shows that, despite the participation in the same publication, they may not maintain sustainable scientific interaction with each other.

The higher percentage of the use of the term “trachoma” as a keyword in publications may be related to the most common use of the name of this disease, besides its availability as a scientific keyword in the main databases for indexing publications, such as the Medical Subject Headings (MeSH, PubMed*)* and the Health Sciences Descriptors (DeCS, by the *Biblioteca Regional de Medicina* [BIREME – Regional Library of Medicine] of the Latin American and Caribbean Center on Health Sciences Information). It may also involve an interest in ensuring greater specificity to the study, as it identifies studies directly related to the disease^16^.

The analyzed publications present the main focuses of research on trachoma in Brazil over time, addressing various aspects about the disease. Historically, during the 1930s and 1940s, trachoma was considered endemic because of its high incidence in the so-called *Dispensários de Tracoma*. From the 1970s on, it ceased to be considered a public health problem, but reemerged around the 1980s among schoolchildren and preschoolers^21^.

As it is a disease strongly related to poverty^22^, trachoma is associated with low income, limited schooling, and inadequate sanitarycondition^1^, which explain it higher incidence in areas with greater social inequality^1^. This aspect reinforces the need to expand the development of epidemiological studies with critical analysis of processes of social determination in previously endemic locations, especially those with low socioeconomic status^1^.

The limitation of studies in Brazil, especially on the spread of *C. trachomatis*, explains the varied detection of cases with the presence of a serotype associated with trachoma in different areas of occurrence of the disease^23^. In past decades, school surveys^10^ and population-based studies^14^ presented prevalence estimates that suggested that trachoma in Brazil^10^ is an important cause of avoidable blindness^2^ and evidence of ophthalmic sequelae of corneal lesions secondary to the disease in indigenous populations^17^.

Women were significantly more affected, when compared them with each other, and active forms were more present among children. On the other hand, cicatricial lesions were more prevalent among adults and older adults^24^, which shows late effects of an active disease that occurred in when younger^24^.

In some regions of Brazil, the low estimated prevalence of the disease, an aspect of national interest, may suggest successful control actions, with timely diagnosis and treatment of cases and contacts to eliminate the disease as a cause of blindness in the country^25^. However, it may present the possibility of undernotification in traditionally endemic areas due to the low sensitivity of the health care and surveillance network in the Brazilian Unified Health System (SUS)^26^. Therefore, the need to strengthen the SUS to expand its responsiveness on neglected topics, such as trachoma, is clear.

The quality of specific treatment monitoring has been a critical issue regarding trachoma control. A study performed in a municipality in the state of Ceará showed a considerable treatment abandonment in the first and second returns, which compromises the control of this disease^27^. Developing studies to expand knowledge about possible failures in the implementation of control actions^28^ and the recognition of its causes is essential. This process is important to strengthen trachoma surveillance and control actions in municipalities in Brazil, especially those considered a high priority^1,21^. Developing epidemiological studies to analyze this public health problem and contribute to governmental agendas^22^ and the use of resources for health actions^29^ and health research and innovation^20^ is fundamental.

The Brazilian Ministry of Health, by Ordinance No. 67 of 2005, established the inclusion of the use of azithromycin, in a single dose at the time of detection, for systemic treatment of cases of trachoma in order to reduce abandonment^30^. Future research may explore the potential effect of availability and access to research funding in different regions of Brazil as a possible influencing factor in scientific production on trachoma.

The limitations of this study were related to the scope of data collection and the indexing process of the Scopus database. Despite the significant scope of this database, with a considerable number of scientific journals and other research publication sites, no database alone includes all journals in which relevant research on trachoma is published. Even with the scope of the literature research in this study, the number of Brazilian bibliographic publications is still small, especially considering population-based prevalence studies in endemic regions and states. Finally, the use of “gray literature” in this study was low. Despite these limitations, the care undertaken in the methodological procedures and the long period analyzed make our analysis and findings considerably robust.

## CONCLUSION

This first scientometric analysis on trachoma in Brazil shows the low development of scientific production on this disease. Although the literature presents a slight increase in it, we observed by the scientometric indicators a higher participation of researchers from outside the areas with greater endemicity and in consolidated Brazilian research centers.

Expanding the funding for research on trachoma in Brazil is important, aiming at mechanisms to ensure access by institutions and researchers from more endemic areas.

The analysis of scientific production on this topic is important to strengthen the development of research and strategic planning of programs for the control of trachoma and neglected tropical diseases in general.
