# Supplementary material for: Scientometric analysis of research on trachoma in Brazil, 2000–2020
Source: Rev Saude Publica. 2022 Nov 18;56:97. doi: 10.11606/s1518-8787.2022056004144 (PMC9749654; doi:10.11606/s1518-8787.2022056004144)
Supplement: Translation

# Translation: Análise cienciométrica da pesquisa sobre tracoma no Brasil, 2000–2020

**Authors:** Adjoane Maurício Silva Maciel, Alberto Novaes Ramos, Anderson Fuentes Ferreira, Nádia Maria Girão Saraiva de Almeida, Vivian da Silva Gomes, Daniela Vaz Ferreira Gómez, Roberto da Justa Pires

**Affiliations:** I Fortaleza CE Brasil Universidade Federal do Ceará. Faculdade de Medicina. Programa de Pós-Graduação em Saúde Pública. Fortaleza, CE, Brasil; II Russas CE Brasil Secretaria Municipal de Saúde. Russas, CE, Brasil; III Fortaleza CE Brasil Universidade Federal do Ceará. Faculdade de Medicina. Departamento de Saúde Comunitária. Fortaleza, CE, Brasil; IV Fortaleza CE Brasil Universidade Estadual do Ceará. Mestrado Profissional em Saúde da Criança e do Adolescente. Fortaleza, CE, Brasil; V Fortaleza CE Brasil Secretaria de Saúde do Estado do Ceará. Fortaleza, CE, Brasil; VI Brasília DF Brasil Ministério da Saúde. Secretaria de Vigilância em Saúde. Brasília, DF, Brasil

**Keywords:** Tracoma, Publicações Científicas e Técnicas, Bases de Dados de Citações, Bibliometria, Indicadores Bibliométricos

## Abstract

**OBJETIVO:**

Analisar o perfil cienciométrico das pesquisas sobre tracoma no Brasil.

**MÉTODOS:**

Pesquisa bibliográfica de publicações sobre tracoma no Brasil indexadas pela base de dados da Scopus, a partir de critérios específicos no período de 2000 a 2020. Foram extraídos e analisados dados sobre autoria, país de origem, instituições e descritores, com análises de tendências temporais. As redes bibliográficas foram construídas via software de visualização cienciométrica *VOSviewer*^®^ 1.6.16.

**RESULTADOS:**

Do total de 42 publicações analisadas sobre tracoma no Brasil, observou-se média anual de dois artigos, com incremento de aproximadamente 50% no período. Verificou-se média de três autores por documento e os inquéritos escolares foram a categoria de assunto mais comum. Os artigos publicados provêm majoritariamente de instituições brasileiras (95,2%), principalmente das sediadas nas regiões Sudeste e Norte. Dez autores mais produtivos estão mencionados nas primeiras autorias em 26,2% (11/42) das publicações e as instituições predominantes estão afiliadas ao estado de São Paulo. O termo *Trachoma* (n = 18) apresenta maior recorrência como descritor.

**CONCLUSÃO:**

Esta primeira análise cienciométrica sobre tracoma no Brasil evidencia limitado número de pesquisas sobre essa doença. Há discreto incremento da produção científica, apesar da concentração da origem em áreas geográficas com menor endemicidade da doença. Maiores investimentos são necessários para o melhor entendimento e controle dessa doença tropical negligenciada. A análise da produção bibliográfica tem papel relevante para fortalecimento do desenvolvimento de pesquisas e planejamento estratégico de programas para o controle de tracoma e doenças tropicais negligenciadas em geral.

## INTRODUÇÃO

O tracoma é uma doença tropical negligenciada (DTN)^1^ causada pela bactéria *Chlamydia trachomatis*, com implicações críticas à saúde das pessoas pela ceratoconjuntivite crônica e recidivante associada^2,3^. Representa no mundo a principal causa de cegueira de origem infecciosa^1^.

Sua elevada carga de morbidade está associada à pobreza em diferentes contextos de vulnerabilidade, como baixo grau de escolaridade, falhas de saneamento básico e higiene, além de restrições de acesso a serviços de saúde^1^. Sua ocorrência, portanto, restringe a perspectiva de uma saúde ocular com expectativa maximizada, em termos de bem-estar em geral, capacidade funcional, inclusão social e qualidade de vida^4^.

Estimativas do ano de 2019 demonstraram que 142,2 milhões de pessoas viviam em áreas endêmicas para tracoma no mundo^3^, com 1,9 milhões sob risco de evolução para deficiência visual ou cegueira irreversível^5^.

Até 2020, nove países no mundo mantinham validada a eliminação do tracoma como problema de saúde pública^6^. Em pelo menos 44 países a doença é reconhecida como problema de saúde pública. Na América Latina, a doença persiste em diferentes áreas do Brasil, Colômbia, Guatemala e Peru, com eliminação validada no México^1^.

A etapa 1 do Inquérito para Validação da Eliminação do Tracoma como Problema de Saúde Pública, desenvolvido no Brasil no período de 2018–2019, evidenciou em áreas não indígenas prevalência abaixo de 5% da forma ativa da doença (tracoma folicular) entre crianças de um a nove anos de idade. A forma sequelar da doença (triquíase tracomatosa), desconhecida pelo sistema de saúde, foi inferior ao valor crítico de 0,2%, com exceção a Unidade de Avaliação do Nordeste Cearense, que apresentou prevalência de 0,22%^7^, no entanto, com valor contido no intervalo de confiança.

Alinhada à agenda para alcance dos Objetivos do Desenvolvimento Sustentável (ODS), a Organização Mundial da Saúde propõe, como uma das metas específicas para DTN no período de 2021–2030, a eliminação do tracoma como problema de saúde pública no mundo^8^. Ressalta-se que a saúde ocular é reconhecida como essencial para alcançar muitos dos ODS^4^.

Apesar de o Brasil manter-se como área prioritária para o controle do tracoma^1^, ainda são limitadas as publicações científicas considerando o contexto epidemiológico e seus diferentes padrões de endemicidade em estados e municípios no país^1^. Torna-se estratégico o reconhecimento de padrões de produção de estudos sobre tracoma no país com vistas a dar suporte a pesquisadores, governos e agências financiadoras para a tomada de decisões mais eficientes.

Análises cienciométricas podem delinear padrões de produção científica e apoiar pesquisadores, governos e agências financiadoras no reconhecimento de áreas e temas com pouco investimento, como o tracoma, para a tomada de decisões mais eficientes. Trata-se de método de pesquisa com tendência de uso cada vez maior, inclusive no Brasil, possibilitado o reconhecimento dos esforços de pesquisa a partir da descrição quantitativa de documentos^9^, a colaboração científica e a caracterização das redes sociais científicas relacionadas a um tema^10^.

Nessas perspectivas, propõe-se neste estudo desenvolver a primeira análise cienciométrica sobre tracoma no Brasil com objetivo de analisar o perfil das pesquisas sobre o tema no período de 2000 a 2020, com foco em gerar subsídios para a proposição de ações e políticas de pesquisa, vigilância e controle do tracoma no país.

## MÉTODOS

Trata-se de pesquisa cienciométrica sobre publicações relativas ao tracoma no Brasil, com produções científicas realizadas no país ou com dados epidemiológicos referentes à doença.

Os dados foram extraídos em julho de 2021 considerando-se todas as publicações do período de janeiro de 2000 a dezembro de 2020, o que totaliza uma série de 21 anos de análise.

Foram recuperadas produções científicas indexadas nas bases de dados Scopus (https://www.scopus.com/home.uri), PubMed (https://pubmed.ncbi.nlm.nih.gov/) e Web of Science (https://mjl.clarivate.com/search-results), por meio de acesso à Comunidade Acadêmica Federada da Coordenação de Aperfeiçoamento de Pessoal de Nível Superior (CAFe-Capes), além da base Dimensions (https://app.dimensions.ai/discover/publication), todas com compatibilidade analítica no software de visualização cienciométrica *VOSviewer*^®^ 1.6.16 (https://www.vosviewer.com/). Esse software pode ser usado para construir uma rede de conhecimento científico, além de exibir estrutura, evolução e cooperação em pesquisa^11^.

A partir de critérios específicos foi realizada busca avançada com aplicação dos descritores, nas bases de dados Scopus: *((AUTHKEY (“Trachoma”) OR TITLE (“Trachoma”) OR ABS (“Trachoma”)) and (AUTHKEY (“Brazil”) OR TITLE (“Brazil”) OR ABS (“Brazil”)) OR (AUTHKEY (“Tracoma”) OR TITLE (“Tracoma”) OR ABS (“Tracoma”)) OR (AUTHKEY (“Brasil”) OR TITLE (“Brasil”) OR ABS (“Brasil”)));* PubMed*: (((tracoma[MeSH Terms]) OR (Tracoma[Title])) OR (Tracoma[Title/Abstract])) AND (((Brasil[MeSH Terms]) OR (Brasil[Title])) OR (Brasil[Title/Abstract]))) OR (((trachoma[MeSH Terms]) OR (Trachoma[Title])) OR (Trachoma[Title/Abstract])) AND (((Brazil[MeSH Terms]) OR (Brazil[Title])) OR (Brazil[Title/Abstract])));* Web of Science*: ((TI=(trachoma) OR AB=(trachoma) OR AK=(trachoma)) AND (TI=(Brazil) OR AB=(Brazil) OR AK=(Brazil))) OR ((TI=(tracoma) OR AB=(tracoma) OR AK=(tracoma)) AND (TI=(Brasil) OR AB=(Brasil) OR AK=(Brasil)))]*; e Dimensions*: (trachoma and Brazil) OR (tracoma and Brasil)]*.

Para análise de indicadores e relações cienciométricas foi selecionada a base de dados Scopus, em virtude de maior número de documentos recuperados, disponibilidade de variáveis e das especificidades do *VOSviewer*^®^. Entretanto, verificou-se restrição em determinados períodos de tempo por conta de questões relativas à indexação de alguns importantes periódicos nacionais e internacionais, além de opiniões de especialistas.

Foram utilizados indicadores de análise cienciométrica relativos à *coautoria* para as unidades: *autor (co-authorship versus author), instituição (co-authorship versus organizations)* e *país* (*co-authorship versus countries*); e à *co-ocorrência* para unidade *descritores próprios do autor (co-occurrence versus authorkeywords)*, seguindo parâmetros de limite máximo de ‘25’ e mínimo de ‘1’ para os itens de visualização cienciométrica.

Na unidade de análise *autor* processou-se a verificação de autores dos estudos selecionados para o período, enquanto *instituição* relaciona-se à afiliação correspondente a cada autor e *país* refere-se à nacionalidade das instituições dos autores das produções científicas. Para a apresentação dos resultados, foram destacadas e apresentadas as dez maiores ocorrências de cada unidade de análise.

Para estruturação de redes bibliográficas foram consideradas as especificidades das referências associadas a cada registro, juntamente com os dados de descritores do autor, com disposição dos termos mais frequentes nas publicações e análise das relações (*clustering*) por meio do *VOSviewer*^®^^11^.

Procedeu-se à visualização de imagens representativas das relações (mapas) entre *autores*, *países*, *instituições* e *descritores* (nós) e a força entre essas relações (espessura dos arcos), além do número de suas contribuições totais (tamanho do nó). Aplicou-se ainda o tesauro para consolidação dos termos analisados.

A pesquisa foi aprovada pelo Comitê de Ética do Hospital São José de Doenças Infecciosas (HSJ) / Secretaria de Saúde do Estado do Ceará sob Parecer Consubstanciado número 3.634.979 (CAAE 18663119.7.0000.5044).

## RESULTADOS

### Literatura Científica, Distribuição Espacial e Tendência Temporal

Inicialmente foram identificados e selecionados, após exclusão de duplicidades, trabalhos científicos na base de dados Scopus (identificados: 59, selecionados: 42), Dimensions (identificados: 52, selecionados: 44), PubMed (identificados: 34, selecionados: 29) e Web of Science (identificados: 31, selecionados: 26) no período do estudo. Reconheceram-se 52 publicações em comum a todas as bases de dados (Tabela 1).

**Tabela 1:** Caracterização dos estudos sobre tracoma segundo título, autoria, ano de publicação, local do estudo e base de dados. Brasil, 2000–2020, (n = 52).

n	Título	Autor	Ano de Publicação	Local do Estudo	Bases de dados			
					Scopus^a^	Dimensions	PubMed	Web of Science
1	*Chlamydia trachomatis serotype A infections in the Amazon region of Brazil: prevalence, entry and dissemination*	Ishaket al.^23^	2015	Maranhão, Pará e Amapá (estados)	X	X	X	X
2	*Community-based transconjunctival marginal rotation for cicatricial trachoma in Indians from the Upper Rio Negro basin*	Soares et al.^17^	2004	Região Alto Rio Negro - Amazonas	X	X	X	X
3	*Corneal findings not related to entropion or trichiasis after trachoma*	Chaves et al.	2001	São Paulo (estado)	X			
4	*Diseases Neglected by the Media in Espírito Santo, Brazil in 2011–2012*	Cavaca et al.^22^	2016	Espírito Santo (estado)	X	X	X	X
5	*Elaboration and validity and reliability analysis of a questionnaire to assess the knowledge of primary care physicians and nurses about trachoma*	Silva et al.	2020	-	X			
6	*Epidemiologic study of trachoma in a community of “Chapada do Araripe”, Pernambuco State - Brazil*	Lucena et al.	2004	Ipubi - Pernambuco	X			
7	*Epidemiological study of trachoma (letter)*	Mörschbächer et al.	2011	-	X			
8	*Epidemiology of trachoma in the village of Araripe plateau - Ceará State*	Lucena et al.^24^	2010	Porteiras - Ceará	X	X	X	X
9	*Factors associated with trachoma in a low-endemic area in southeast Brazil*	D’Amaral et al.	2005	São Paulo - São Paulo	X	X	X	
10	*Factors associated with trachoma treatment and control treatment in schools of municipality of the Northeast Region, Brazil*	Maciel et al.^27^	2020	Russas - Ceará	X	X	X	
11	*Household Survey of Trachoma among Children Living in Pernambuco, Brazil*	Brito et al.^14^	2019	Pernambuco (estado)	X	X	X	X
12	*It was urgent and indispensable to act: The trachoma in São Paulo in the early twentieth century*	Lódola et al.	2019	-	X			
13	*Laboratory diagnosis of trachoma in Serrolândia village of Ipubi Town, Pernambuco – Brazil*	Lucena et al.	2005	Ipubi - Pernambuco	X			
14	*Preliminary evidence that synanthropic flies contribute to the transmission of trachoma causing Chlamydia trachomatis in Latin America*	Reilly et al.	2007	Ilha de Marajó - Pará	X	X	X	X
15	*Prevalence and spatial distribution of trachoma among schoolchildren in Botucatu, São Paulo – Brazil*	Schellini et al.	2010	Botucatu - São Paulo	X	X	X	X
16	*Prevalence of infection by Chlamydia trachomatis in ocular samples of patients with conjunctivitis in genetic and molecular biology laboratory from metropolitan area of Florianópolis, Brazil*	Machado et al.	2009	Florianópolis - Santa Catarina	X			X
17	*Prevalence of trachoma and associated factors in students from the Jequitinhonha Valley, Minas Gerais, Brazil*	Silva et al.	2020	Vale do Jequitinhonha - Minas Gerais	X	X	X	X
18	*Prevalence of trachoma among schoolchildren in Bauru - São Paulo State, Brazil*	Ferraz et al.	2010	Bauru - São Paulo	X	X	X	X
19	*Prevalence of trachoma in a population of the upper Rio Negro basin and risk factors for active disease*	Cruz et al.	2008	São Gabriel da Cachoeira - Amazonas	X	X	X	X
20	*Prevalence of trachoma in Botucatu city - Sao Paulo state* [Prevalência de tracoma cicatricial em Botucatu - Estado de São Paulo]	Schellini et al.	2006	Botucatu - São Paulo	X			
21	*Prevalence of trachoma in Brazilian schoolchildren*	Lopes et al.	2013	Brasil (municípios)	X	X	X	X
22	*Prevalence of trachoma in preschool and schoolchildren in the city of São Paulo*	Koizumi et al.	2005	São Paulo - São Paulo	X	X	X	
23	*Prevalence of trachoma in schoolchildren in Brazil*	Luna et al.	2016	Brasil (municípios)	X	X	X	X
24	*Prevalence of trachoma in schoolchildren in the Marajo Archipelago, Brazilian Amazon, and the impact of the introduction of educational and preventive measures on the disease over eight years*	Favacho et al.	2018	Ilha de Marajó - Pará	X	X	X	X
25	*Prophylaxis and treatment of diseases in western São Paulo state: the Sanitation Service and trachoma in the early twentieth century*	Lodola et al.	2020	-	X	X	X	
26	*Sequelae from Epidemic Viral Conjunctivitis Can Be Associated with Inflammatory Trachoma in Schoolchildren?*	Meneghim et al.	2016	Botucatu - São Paulo	X	X	X	X
27	*Spatial distribution of trachoma cases in the City of Bauru, State of São Paulo, Brazil, detected in 2006: defining key areas for improvement of health resources*	Macharelli et al.^29^	2013	Bauru - São Paulo	X	X	X	X
28	*Survey of trachoma within school students in the state of Roraima, Brazil*	Medina et al.	2011	Roraima (estado)	X	X	X	X
29	*Trachoma among the Yanomami Indians*	Paula et al.	2002	Região Alto Rio Negro - Amazonas	X	X	X	X
30	*Trachoma and corneal diseases among Indians of the Alto Rio Negro, Amazonas, Brazil*	Reis et al.	2002	Região Alto Rio Negro - Amazonas	X	X		
31	*Trachoma and ethnic diversity in the Upper Rio Negro Basin of Amazonas State, Brazil*	Alves et al.	2002	Região Alto Rio Negro - Amazonas	X	X	X	X
32	*Tracoma: de lo básico a lo clínico*	Carvajal-Fernández et al.	2017	-	X			X
33	*Trachoma: Epidemiologic study of scholars from Alagoas state-Brazil*	Damasceno et al.	2009	Alagoas (estado)	X	X	X	X
34	*Trachoma epidemiological school survey in the city of Embu das Artes – SP*	Caninéo et al.	2012	Embu das Artes - São Paulo	X	X	X	
35	*Trachoma elimination in Latin America: prioritization of municipalities for surveillance activities*	Saboyá-Díaz et al.^1^	2019	América Latina	X	X	X	X
36	*Trachoma in Indigenous Settlements in Brazil, 2000–2008*	Freitas et al.	2016	Brasil	X	X	X	X
37	*Trachoma in patients with allergic conjunctivitis*	Bezerra et al.	2010	João Pessoa - Paraíba	X			
38	*Trachoma in schoolchildren of the city of Botucatu, Sao Paulo, Brazil: detection and health promotion of a neglected disease*	Meneghim et al.^25^	2016	Botucatu - São Paulo	X			
39	*Trachoma prevalence among schoolchildren in the municipality of Turmalina, Minas Gerais state*	Silva et al.	2016	Turmalina - Minas Gerais	X			
40	*Trachoma prevalence and risk factors among preschool children in a central area of the city of São Paulo, Brazil*	Caligaris et al.	2006	São Paulo - São Paulo	X	X	X	X
41	*Trachoma prevalence in preschoolers and schoolchildren in Botucatu, São Paulo State, Brazil,1992*	Medina et al.^21^	2002	Botucatu -São Paulo	X	X	X	
42	*Trachoma: Still being an important blinding disease (review)*	Schellini et al.	2012	-	X			X
43	*Analysis of interventions and socio environmental factors associated with the occurrence of trachoma in Pernambuco in two surveys on schoolchildren conducted in 2006 and 2012*	Alves et al.	2016	Pernambuco (municípios)		X		
44	*Blinding trachoma among Maku Indians of the upper Rio Negro: a neglected public health problem*	Cruz et al.	2017	Região Alto Rio Negro -Amazonas			X	
45	*Elimination of Trachoma as cause of blindness in Itapevi, State of Sao Paulo, Brazil*	Joseph et al.	2015	Itapevi - São Paulo				X
46	*Epidemiological aspects and prospects of the elimination of Trachoma (2018-2020) as a public health problem in Brazil*	Gómez et al.	2018	Brasil (estados)		X		
47	*Epidemiology and operational aspects of trachoma surveillance and control in a school in the Municipality of São Paulo, Brazil*	Chinen et al.	2006	São Paulo - São Paulo		X		
48	Estratégia eficaz para o enfrentamento do tracoma no Estado do Ceará	Gomes et al.	2019	Fortaleza - Ceará		X		
49	Epidemiological survey about of trachoma in students schools belonging to cities localized in the IV Gerencia Regional de Saude located in the state of Pernambuco, Caruaru –PE.	Germinio et al.	2016	Caruaru - Pernambuco		X		
50	Survey of prevalence trachoma of children in Distrito Federal, Brazil, july/2010	Jesus et al.	2013	Distrito Federal- Brasília		X		
51	Trachoma as cause of blindness: literature review	Silva et al.	2017	-		X		
52	Tracoma: uma antiga patologia ainda negligenciada na atualidade	Silva et al.	2015	-		X		

Fonte: Scopus, PubMed, Web of Science, Dimensions.

a Base de dados Scopus com artigos analisados de n = 1 a n = 42.

Artigo de revisão, editorial (literatura cinza).

Para a análise foram consideradas as 42 produções científicas da base de dados Scopus que apresentavam 138 autores, tendo em vista que o software de análise cienciométrica *(VOSviewer*^®^*)* processa em sua rotina uma base (Tabela 1). Verificou-se que a tipologia de ‘artigo original’ foi a principal modalidade de publicação, representando 95,2% (40/42) de todos os trabalhos analisados (Tabela 1).

Entre os assuntos mais comuns destes trabalhos, inserem-se: inquéritos entre escolares (n = 14), análise em populações indígenas (n = 4), sequelas da doença (n = 4), diagnóstico laboratorial (n = 4), fatores de risco do tracoma (n = 3), ‘literatura cinza’ (n = 6), pesquisas de base populacional (n = 2), distribuição espacial (n = 1), categorização epidemiológica de municípios para fins de vigilância (n = 1) e outros (n = 3).

Verificou-se produção bibliográfica em todas as regiões do país, com incremento geral em 50% nas produções bibliográficas na segunda década (2010–2020) do estudo (Figura 1).

**Figura 1:** Produção bibliográfica sobre tracoma, Brasil, 2000–2020 (n = 42).

Foram identificadas pesquisas sobre tracoma em 13 estados brasileiros: São Paulo (n = 12), Amazonas (n = 5), Pernambuco (n = 3), Ceará (n = 2), Minas Gerais (n = 2), Alagoas (n = 1), Espírito Santo (n = 1), Maranhão, Amapá (n = 1), Pará (n = 3), Paraíba (n = 1), Roraima (n = 1) e Santa Catarina (n = 1). Observou-se ainda representação de dados gerais do Brasil (n = 3) e América Latina (n = 1). Demais trabalhos foram considerados ‘literatura cinza’ (n = 6) (Tabela 1).

### Análise Cienciométrica

A análise cienciométrica sobre tracoma indicou média nacional anual de dois artigos nas duas últimas décadas. Foi mantida média de três autores por documento analisado, com variação de dois a dez autores/autoras para cada publicação (Tabela 1).

Os dez pesquisadores mais produtivos estão mencionados como primeiras autorias em 26,2% (11/42) das publicações, com maiores contribuições evidenciadas para Medina N.H., participante em 35,7% (15/42) das produções científicas. Cruz A.A.V. é, proporcionalmente, o autor mais citado (Tabela 2). Na relação *co-authorship versus author* destaca-se a produtividade de Medina N.H. (*cluster* azul) e conexão com demais *clusters*, seguida pela rede de produção científica dos autores Schellini S.A. (*cluster* rosa), Cruz A.A.V. (*cluster* verde) e Cardoso M.R.A. (*cluster* azul) (Figura 2A).

**Figura 2:** Relação de produção bibliográfica em tracoma, Brasil, 2000–2020 (n = 42).

**Tabela 2:** Produção bibliográfica sobre tracoma segundo autoria e país. Brasil, 2000–2020, (n = 42).

Produção bibliográfica	Número de publicações	Centralidade de intermediação		% Produtividade
		Citações	Força de ligação	
Autoria (Open Researcher and Contributor ID [ORCiD])				
Medina, N.H. (0000-0002-6544-6674)	15	142	70	35,7
Schellini, S.A. (0000-0002-6938-1230)	7	20	23	16,7
Cruz, A.A.V. (0000-0002-8972-5571)	6	77	14	14,2
Cardoso, M.R.A. (0000-0001-6092-9215)	6	47	34	14,2
Lopes, M.F.C. (0000-0002-5392-1001)	5	28	37	11,9
Luna, E.J.A. (0000-0002-1145-9672)	5	27	30	11,9
Padovani, C.R. (0000-0002-7719-9682)	5	12	19	11,9
Koizumi, I.K. (0000-0001-5602-4878)	4	30	26	9,5
Caligaris, L.S.A. (não encontrado)	3	29	19	7,1
Favacho, J. (0000-0001-7926-7952)	3	23	14	7,1
País (Instituição)				
Brasil	40	211	3	95,2
Reino Unido	1	12	1	2,4
Austrália	1	4	1	2,4
México	1	1	1	2,4
Estados Unidos da América	1	1	1	2,4
Arábia Saudita	1	0	1	2,4
Colômbia	1	0	0	2,4

Fonte: *VOSviewer*^®^.

Os estudos recuperados sobre o tracoma no Brasil foram publicados a partir de instituições oriundas de sete países. Foram identificadas publicações de autores afiliados a instituições no Brasil (n = 40) e simultaneamente ao Reino Unido (n = 1), Austrália (n = 1) e Arábia Saudita (n = 1). As publicações de autores exclusivamente fora do país tiveram como origem instituições dos Estados Unidos da América e México (n = 1) e outro da Colômbia (n = 1) (Tabela 2). A análise de *co-authorship versus countries* de instituições indica a relação de publicações entre instituições dos seguintes continentes: Américas (n = 4), Europa (n = 1), Ásia (n = 1) e Oceania (n = 1) (Figura 2B).

No total, 55 instituições participaram das pesquisas selecionadas, com predomínio entre as dez mais produtivas daquelas sediadas no estado de São Paulo (6/10 – 60%), região Sudeste do Brasil, e participação mais expressiva da Secretaria de Saúde do Estado de São Paulo (33,3%) e da Universidade de São Paulo (USP) (26,2%) (Tabela 3). A relação *co-authorship versus organizations* coloca em evidência a relação entre instituições das regiões Sudeste (São Paulo) e Norte (Amazonas), além do Ministério da Saúde que atua como ponto de conexão com outros estados (Figura 2C).

**Tabela 3:** Produção bibliográfica sobre tracoma segundo instituições, Brasil, 2000–2020, (n = 42).

Instituições	Publicações	% de publicações	Citações
Secretaria do Estado da Saúde de São Paulo, São Paulo, São Paulo	14	33,3	112
Universidade de São Paulo (USP), São Paulo, São Paulo	11	26,2	80
Universidade Estadual Paulista (UNESP), Botucatu, São Paulo	7	16,7	20
Universidade de São Paulo (USP), Ribeirão Preto, São Paulo	5	11,9	51
Ministério da Saúde do Brasil, Brasília, Distrito Federal	5	11,9	28
Instituto Evandro Chagas, Belém, Pará	3	7,1	23
Universidade Federal de São Paulo (UNIFESP), São Paulo, São Paulo	3	7,1	16
Universidade Estadual de Montes Claros, Montes Claros, Minas Gerais	3	7,1	1
Universidade Federal dos Vales do Jequitinhonha e Mucuri, Diamantina, Minas Gerais	3	7,1	1
Secretaria Municipal de Saúde de São Paulo, São Paulo	2	4,8	23

Fonte: *VOSviewer*^®^.

Nota: seleção/classificação: 1. Número de publicações; 2. Citações.

Foram reconhecidos dez descritores mais frequentemente citados nas publicações científicas analisadas. O termo Tracoma (n = 18) apresenta maior recorrência como descritor sendo duas vezes mais frequente do que “*Chlamydia trachomatis*” (n = 8) e “Tracoma/epidemiologia” (n = 7) (Figura 1). A análise *co-occurrence versus author keywords* evidencia maior relação entre esses descritores citados (Figura 2D).

## DISCUSSÃO

Esta pesquisa inédita comprova o limitado número de estudos sobre o tracoma no Brasil e revela a concentração de pesquisadores e centros localizados fora das principais áreas endêmicas do país. A despeito da tendência de aumento verificada na última década, persistem lacunas críticas que reiteram o caráter de doença negligenciada^9^, não apenas por falhas da ciência, mas também por falhas de políticas públicas^12^.

Apesar de o Projeto de Mapeamento Global para Tracoma propor a condução de pesquisas em todo o mundo para definição do mapa de linha de base do tracoma em países endêmicos^13^, o número insuficiente de pesquisas sobre o tracoma no Brasil reduz o reconhecimento da doença, assim como o planejamento e implementação de ações de controle^14^. Esse contexto indica indiretamente baixa priorização de investimentos em pesquisas sobre o tema para superar essa condição crônica nos países endêmicos^15^ demonstrada por meio das limitações evidenciadas por este estudo.

O Brasil representa uma reconhecida liderança científica em temas de Medicina Tropical, particularmente na América Latina, com notável contribuição em diversas DTN^1^, entretanto, há limitação de produções sobre temas ligados ao tracoma.

A saúde e a visão oculares têm implicações gerais importantes e profundas para diversas dimensões da vida, saúde, desenvolvimento sustentável e economia^4^. Esses aspectos dificultam decisivamente o caminho para alcance dos ODS, especificamente da meta de eliminação da doença até 2030. Reforçam a necessidade de se reiterar a responsabilidade do Brasil, como país endêmico, em garantir investimentos adicionais para intensificação de ações de vigilância e controle^15^ e em estabelecer estratégias com base em parcerias consistentes para desenvolvimento tecnológico e científico^16^.

Assim, a saúde ocular é essencial para alcançar muitos dos ODS em 2030. Desde os anos 1990, a prevalência estimada da oncocercose e do tracoma, as grandes causas infecciosas de cegueira, tem diminuído de modo significativo. Há uma expectativa de que até 2030 a transmissão da oncocercose seja finalmente interrompida e de que o tracoma seja eliminado como um problema de saúde pública em todos os países do mundo^4^.

Há significativa concentração de produção científica em tracoma nas regiões Norte e Sudeste do Brasil, de modo que os estados de São Paulo e Amazonas são as unidades federativas mais destacadas. Essa distribuição, no que tange à região Norte do país, pode estar vinculada à produção de pesquisas com populações indígenas, em contextos endêmicos para tracoma^17^.

Entretanto, as pesquisas nessa região foram mais frequentes na década passada, o que destaca a necessidade de se sustentar a capacidade de desenvolvimento de pesquisas voltadas para populações mais vulneráveis de áreas endêmicas^15^.

No caso da região Sudeste, destacam-se as instituições mais participativas na produção bibliográfica, especialmente aquelas com base no estado de São Paulo, situação provavelmente associada à maior capacidade destas instituições de captação de recursos, infraestrutura e desenvolvimento de pesquisas^18^. Pode-se considerar ainda a capacidade de composição de cooperações técnico-científicas entre grupos de pesquisadores do país e do exterior^19^.

A distribuição da origem das publicações evidencia um crítico contraste na produção relacionada ao tracoma em instituições localizadas em áreas geográficas com menor endemicidade da doença^16^. Ressalta-se que a existência de pesquisas está relacionada diretamente com desenvolvimento humano e social. Além disso, registra-se a limitada disponibilização de acesso a financiamento específico para pesquisas em DTN, particularmente aquelas mais negligenciadas, como o tracoma^15^.

Outro aspecto importante é a força de mobilização de autores para uma temática, assim como a colaboração entre eles, elementos indispensáveis para a capacidade de produção científica^20^. Entretanto, em quase duas décadas desta análise verificou-se discreto aumento da média de autores por pesquisas em tracoma, o que reforça o caráter de não priorização. O maior investimento em cooperação científica tem o potencial de ampliar a integração com instituições em áreas que apresentam menor desenvolvimento de pesquisa^20^. Ressalta-se, entretanto, que esse processo pode causar dependência na definição de temas, sem consideração às necessidades dos locais onde a doença tem maior impacto^16^.

Reconheceu-se que pesquisadores com primeira posição na autoria demonstraram atribuição importante na conexão de colaboração entre demais autores. Além de uma considerável produção científica, esses pesquisadores estabelecem vínculos importantes e cooperação na condução de estudos em instituições com possível acesso a financiamentos para pesquisas^16^. Supõe-se, dessa forma, que atuam como importante elo para construção das redes bibliográficas verificadas neste estudo^10^. Apesar do incremento, a cooperação entre pesquisadores sobre tracoma no Brasil ainda revela relativo distanciamento entre autores de estudos não vinculados a instituições com afiliação mais recorrente nas publicações e coautorias. Evidencia também que, apesar da participação em uma mesma publicação, podem não manter interação científica sustentável entre si.

A ocorrência do termo *Trachoma* como descritor em maior percentual de publicações pode estar relacionado ao uso mais comum da nomenclatura da doença, além da disponibilidade como termo científico descritor nas principais bases de dados de indexação de publicações, como o *Medical Subject Headings (*Mesh, PubMed*)* e o Descritores em Ciências da Saúde (Decs, pela Biblioteca Regional de Medicina [Bireme], do Centro Latino-Americano e do Caribe de Informação em Ciências da Saúde). Pode refletir ainda um interesse de garantir maior especificidade ao trabalho, ao identificar pesquisas relacionadas diretamente à doença^16^.

Outro aspecto importante identificado é que as publicações analisadas demonstram os principais focos da pesquisa em tracoma no Brasil ao longo do tempo, abordando aspectos diversos sobre a doença. Historicamente, nas décadas de 1930 e 1940 o tracoma era considerado endêmico, com alta incidência nos chamados Dispensários de Tracoma. A partir da década de 1970, deixou de ser considerado como problema de saúde pública, mas reemergiu por volta dos anos 1980 entre escolares e pré-escolares^21^.

Doença fortemente relacionada à pobreza^22^, é reconhecidamente associada à baixa renda, limitada escolaridade e inadequada condição sanitária^1^, o que justifica sua expressão principalmente em áreas com maior desigualdade social^1^. Esse aspecto reforça a necessidade de se ampliar o desenvolvimento de estudos epidemiológicos em locais anteriormente endêmicos, especialmente aqueles com baixo nível socioeconômico, com análise crítica de processos de determinação social^1^.

A limitação de estudos no Brasil, especialmente sobre a disseminação de *C. trachomatis*, justifica a variação de detecção de casos com presença de sorotipo associado a tracoma em diferentes áreas de ocorrência da doença^23^. As estimativas de prevalência revelada em décadas passadas a partir de inquéritos escolares^10^ e de estudo de base populacional^14^ sugerem a importância do tracoma no país^10^, inclusive como importante causa de cegueira evitável^2^, além da evidência de sequelas oftalmológicas por lesões corneanas secundárias à doença em populações indígenas^17^.

As mulheres foram significativamente mais afetadas comparativamente e as formas ativas foram mais presentes entre crianças. Por outro lado, as lesões cicatriciais foram mais prevalentes entre adultos e idosos^24^, o que indica efeitos tardios de uma doença ativa que ocorreu em faixas etárias mais jovens^24^.

Em algumas regiões do país, a baixa prevalência estimada da doença, considerada de interesse nacional, pode sugerir ações exitosas de controle, com diagnóstico e tratamento oportuno de casos e comunicantes para eliminação da doença como causa de cegueira no país^25^. Entretanto, pode indicar a possibilidade de subnotificação em áreas tradicionalmente endêmicas, por baixa sensibilidade da rede de atenção e vigilância em saúde no Sistema Único de Saúde (SUS)^26^. Portanto, há uma clara necessidade de fortalecimento do SUS para ampliar a capacidade de resposta sobre temas negligenciados, como o tracoma.

Uma questão crítica apontada para o controle do tracoma tem sido a qualidade do monitoramento do tratamento específico. Estudo realizado em município do estado do Ceará evidenciou registro considerável de abandono do tratamento no primeiro e segundo retornos, o que compromete o controle da doença^27^. Torna-se fundamental o desenvolvimento de estudos para ampliar o conhecimento sobre as possíveis falhas na implementação das ações de controle^28^, com reconhecimento das causas. Ressalta-se a importância desse processo para o fortalecimento das ações de vigilância e controle do tracoma em municípios no Brasil, especialmente aquelas consideradas de alta prioridade^1,21^. Também é essencial o desenvolvimento de estudos epidemiológicos para identificação desse problema de saúde pública com vistas a contribuir para a construção de agendas públicas governamentais^22^ assim como de utilização de recursos destinados às ações em saúde^29^, mas também em pesquisa e inovação em saúde^20^.

O Ministério da Saúde, por meio da Portaria n° 67 de 2005, determinou a inclusão do uso da azitromicina para tratamento sistêmico dos casos de tracoma, em dose única no momento da detecção, a fim de reduzir a problemática do abandono^30^. Pesquisas futuras poderão explorar o potencial impacto da disponibilidade e do acesso a financiamentos para pesquisa em diferentes áreas do país, como possível fator influenciador na produção sobre tracoma.

As limitações do presente estudo referem-se ao escopo da coleta de dados assim como do processo de indexação da base Scopus. Apesar da significativa abrangência dessa base, com indexação de quantidade considerável de periódicos científicos e de outros locais de publicação de pesquisas, ressalta-se que nenhum banco de dados isoladamente incluirá todos os periódicos nos quais as pesquisas relevantes sobre tracoma são publicadas. Mesmo com a abrangência da pesquisa de literatura empreendida neste estudo, registra-se ainda um número reduzido de produções bibliográficas brasileiras, particularmente de estudos de prevalência de base populacional em regiões e estados endêmicos. Por fim, registramos a baixa utilização de bibliografia do tipo ‘cinzenta’. A despeito dessas limitações, os cuidados empreendidos nos procedimentos metodológicos e o extenso período analisado, trazem considerável robustez às análises realizadas e aos achados apresentados.

## CONCLUSÃO

Este trabalho revela, a partir da primeira análise cienciométrica sobre tracoma no Brasil, o baixo desenvolvimento de pesquisas científicas sobre a doença. Embora a literatura indique discreto incremento da produção, por meio dos indicadores cienciométricos analisados, registra-se maior participação de pesquisadores fora de áreas com maior endemicidade e em centros consolidados de pesquisa no país.

Ressalta-se ainda a importância de se ampliar o financiamento de pesquisas em tracoma no país, salvaguardando-se mecanismos para garantir o acesso por instituições e pesquisadores presentes em áreas com maior endemicidade.

A análise da produção bibliográfica tem papel relevante para fortalecimento do desenvolvimento de pesquisas e planejamento estratégico de programas voltados ao controle de tracoma e DTN em geral.
